# Open research practices: unintended consequences and suggestions for averting them. (Commentary on the Peer Reviewers' Openness Initiative)

**DOI:** 10.1098/rsos.160109

**Published:** 2016-04-20

**Authors:** D. V. M. Bishop

**Affiliations:** Department of Experimental Psychology, University of Oxford, South Parks Road, Oxford OX1 3UD, UK

**Keywords:** data-sharing, reproducibility, data-dredging, ethics

## Abstract

The Peer Reviewers' Openness Initiative (PROI) is a move to enlist reviewers in the promotion of data-sharing. In this commentary, I discuss objections that can be raised, first to the specific proposals in the PROI, and second to data-sharing in general. I argue that although many objections have strong counter-arguments, others merit more serious consideration. Regarding the PROI, I suggest that it could backfire if editors and authors feel coerced into data-sharing and so may not be the most pragmatic way of encouraging greater openness. More generally, while promoting data-sharing, we need to be sensitive to cases where sharing of data from human participants could create ethical problems. Furthermore, those interested in promoting reproducible science need to defend against an increased risk of data-dredging when large, multivariable datasets are shared. I end with some suggestions to avoid these unintended consequences.

## 1. Introduction

I welcome the move to make science more open by encouraging authors to publish data and materials alongside their research articles [1]. Openness helps ensure analyses are accurate and data are not lost, and it gives value for money by making data available for reuse in meta-analyses or in studies of new questions. In addition, where analysis scripts as well as data are available, this can be an invaluable training resource. I have not, however, signed the Peer Reviewers' Openness Initiative (PROI). I thought long and hard about whether to do so. I support the initiative's aims and values, but I found sufficient problems with it that I decided to abstain. With any new initiative, it is important to consider the costs as well as benefits. My aim here is to discuss possible unintended consequences, first of the specific proposals in the PROI, and second of current systems of data-sharing in general. I am optimistic that problems can be overcome if we anticipate them, and I end with some suggestions that could help assuage the anxieties that some researchers have about data-sharing.

## 2. Consequences specific to the Peer Reviewers' Openness Initiative

The idea behind the PROI is that there is currently little incentive for authors to share their data; peer reviewers could provide some motivation by refusing to proceed with a full review of a research paper unless and until the data and related materials are either made available, or a good reason is provided for why this is not possible.

Let us consider the scenario proposed by Morey *et al.* [1]. Imagine a journal editor processing a new submission. We know that much of the delay in publishing arises because of difficulties finding reviewers, and that editors have to issue numerous invitations to find an adequate number [2]. So the editor notes with relief that Dr Morey agrees to review a manuscript. However, no sooner does she send him the manuscript than it bounces back with a statement that, in accordance with PROI, he cannot complete his review until the data are made available. It is hoped that this will encourage the editor to go back to the author to ask for the data. But unless the data are already curated for publication, this will introduce a delay, the length of which will depend on the type of study and the other commitments and resources of the author. For a relatively simple experiment, it may be possible to document and deposit the data, analysis scripts and materials in a matter of hours, but with more complex datasets, as in bioinformatics, genetics or neuroimaging, this could take weeks [3,4]. Some authors will feel penalized by this, especially if others who submit to the journal are not required to provide their data. We then have to consider the reaction of the editor. I suspect this will vary according to whether he or she is committed to open data and whether or not the journal policy mandates it: if it does, then the editor may regard the reviewer's response as reasonable, though they may still be concerned at imposing a delay in processing the paper—which will impact badly on their journal statistics for publication lag. But many journals do not mandate deposition of data and we know that some editors are not fully committed to data-sharing [5]. A reviewer who demands the data might be seen as usurping the editorial role. It could be said that part of the purpose of PROI is to put pressure on editors. However, they may respond by finding another reviewer, rather than complying with a reviewer request that does not reflect journal policy. If that happens, nothing is gained other than ill will from author and editor.

It will be informative to follow the experience of those adopting the PROI to see how often that happens—this is a novel experiment that is ahead of us and I will follow it with interest. But I am dubious as to whether it will have the desired effect, and concerned it might create more bad feeling about data-sharing than positive change.

The PROI is an idealistic initiative, designed to encourage broader adoption of data-sharing practices. However, any such initiative needs to be pragmatic; making people feel coerced to adopt what, for many, are novel practices may not be the best way to get the academic community on side.

So what can be done? I think the PROI would benefit from a very simple tweak. Peer reviewers could undertake not to review for journals that do not mandate open data—and if asked to do so, they should make clear the reason for refusing. Journal boycotts by peer reviewers are nothing new [6] and this systematic approach would, I suspect, be more effective in changing journal policies than an action that could be seen as delaying the review process for a small subset of authors who were allocated to a reviewer who had signed the PROI. I agree we need to change the culture and incentives to encourage open data, but I think the PROI in its current form risks making authors feel unjustly penalized, and editors feel that reviewers are stepping beyond their remit.

## 3. Consequences of data-sharing for researchers

Anyone who has argued for open data will be aware that there are many researchers who are dubious about the whole idea. I make the case here that many of the arguments against data-sharing do not bear scrutiny, but that there are some risks that need to be managed. Table 1 summarizes four arguments that relate to perceived conflict between the costs and benefits to researchers versus the advancement of science. I will not devote further space to these, as in my view there are good counter-arguments, and they are relatively easy to address with changes in the incentive structure to reward data-sharing.

**Table 1.** Conflict between interests of researchers and advancement of science.

argument	counter-argument
1. Lack of time to curate data.	Unless adequately curated, data will over time become unusable, including by the original researcher [7].
2. Personal investment—reluctance to give data to freeloaders.	Reuse of data increases its value and the researcher benefits from additional citations [8]. There is also an ethical case for maximizing use of data obtained via public funding [9].
3. Concerns about being scooped before the analysis is complete.	This is a common concern though there are few attested cases. A time-limited period of privileged use by the study team can be specified to avoid scooping [10].
4. Fear of errors being found in the data.	Culture change is needed to recognize errors are inevitable in any large dataset [11] and should not be a reason for reputational damage. Data-sharing allows errors to be found and corrected.

## 4. Consequences of data-sharing for human participants

Another set of issues revolves around the rights and protection of human participants. The PROI was received with alarm by some researchers working with patient groups, who were concerned that they would be required to make their data publicly available. In fact, this is not what is proposed: Morey *et al.* [1] noted that there will be circumstances when it is not appropriate to share data because of legal or ethical concerns, and that in such cases all that is required is a clear statement of the reasons for not making data open. However, this raises the question of what are valid reasons.

In the past, data-sharing was often explicitly vetoed in consent forms and information sheets required by ethics committees (known in the US as Institutional Review Boards). For many years, these routinely reassured participants that their data would not be made available to anyone outside the research team. It is now recognized that this can be unduly restrictive, and researchers are encouraged to plan ahead to maximize data usage [10].

Problems may still arise when participants gave consent for a specific analysis, but the data have potential to be used more widely. This would not normally raise difficulties in practice, as most secondary analyses are likely to be innocuous: one cannot imagine participants objecting if, for instance, data from a study on memory were subsequently analysed to look at time-of-day effects. However, if secondary analysts focus on sensitive issues, such as effects related to gender, psychiatric status, political allegiance or race, we need to be aware that, unless participants gave permission for this extension of the analysis, it could be unethical and possibly illegal. This can apply not only to patient data, but also to studies of individual differences in non-patient samples. Where potentially controversial data come from adults who were recruited as children, with parental consent, further ethical issues may be raised.

## 5. Consequences of data-sharing for science

There is one more unintended consequence of data-sharing, which has received less attention than the others. Open data are typically presented as part of the solution to the reproducibility crisis, but there are situations when it could have the opposite effect. Any large, multivariable dataset provides ample opportunities for *p*-hacking—i.e. digging around in the data to look for ‘interesting’ patterns that give significant results.

**Figure 1.**
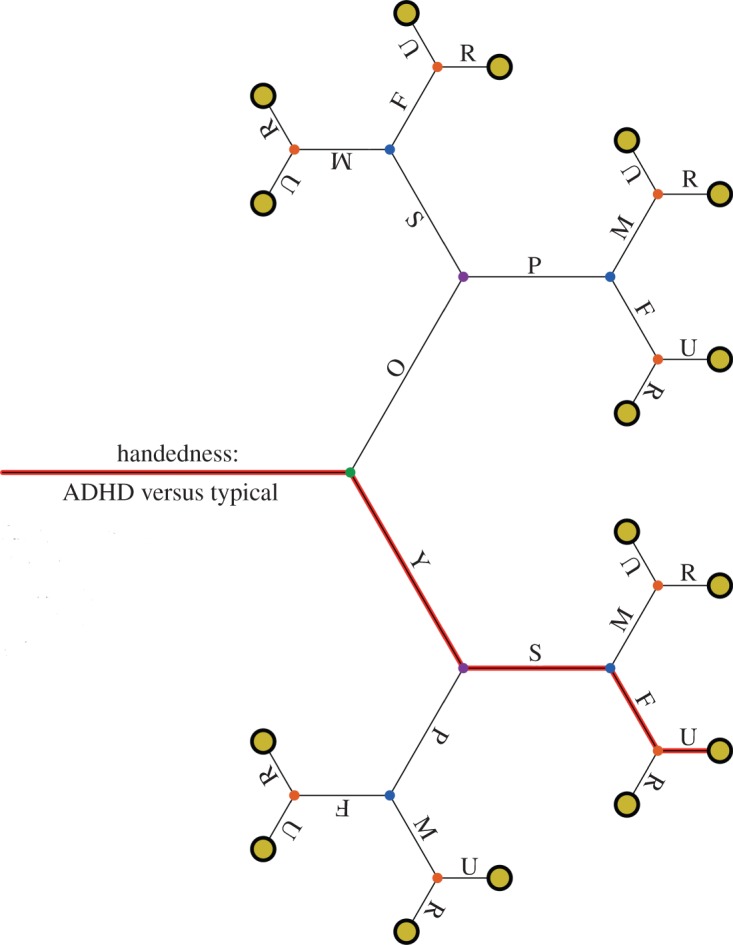
The Garden of Forking Paths: an illustration of how selection of specific measures or subgroups can increase the number of possible comparisons, affecting the likelihood of obtaining a ‘significant’ *p*-value by chance. See text for explanation, or refer https://figshare.com/s/f50547936de09afef9ff for animated version. The figure title is inspired by Gelman & Loken's [13] literary reference. O, Old; Y, Young; S, Hand skill; P, Hand preference; M, male; F, female; U, urban; R, rural.

The way in which analytic flexibility multiplies opportunities for spurious findings is well documented in relation to primary research [12] but we should not forget that it also affects secondary analyses. Figure 1 illustrates how opportunities for false positives can mount up when one has a large dataset and a flexible approach to analysis. Consider an investigator who is interested in testing whether handedness is associated with attention deficit hyperactivity disorder (ADHD), and has access to a large dataset that has measures of both hand skill and hand preference at 6 years and 10 years, as well as demographic information. Suppose further that there is no true association in the population. The green node corresponds to the two-group comparison, where probability of obtaining a *p*-value less than 0.05 is 1 in 20. An investigator who compares ADHD (A) and typical (T) children (green node) may be disappointed to find the comparison is non-significant. But the investigator may be tempted to do a subgroup analysis, because the association looks different in older (O) and younger children (Y) (purple nodes). He might then realize that results vary for measures of hand skill (S) versus hand preference (P) (blue nodes). He could then decide to subdivide the sample by gender (M versus F) (orange nodes) and according to whether children are from urban (U) rather than rural (R) areas (khaki nodes). If all these combinations of possibilities were to be considered, the chance of a finding at least one ‘significant’ result rises to 1−0.95^16^ = 0.56. When doing numerous comparisons, there are legitimate methods for adjusting *p*-values to guard against false positives, but in practice these are often ignored. It is particularly problematic if researchers focus on reporting only the ‘significant’ *p*-values. The animated version of figure 1 shows how interpretation of *p*-values becomes distorted if researchers ignore the paths that lead to non-significant results [13]. If the researcher presents what looks like a simple two-group comparison (e.g. ADHD and typical young, urban females differ on a measure of relative hand skill), which is in reality selected from a wide range of possible contrasts, then uncorrected *p*-values can be highly misleading.

A related problem concerns publication bias. Suppose we have a researcher interested in the possible impact of diet on children's cognitive development. There are several large publicly available datasets that could be used to address this question. If a positive association is found, then the result is reported, but if it is not, the researcher is likely to move on to look at something else—unless they embarked on the analysis with the intent of disproving the link. This kind of publication bias happens with primary analyses of data, but the motivation for ignoring ‘uninteresting’ findings is enhanced if the analyst did not have the cost of data collection.

When unconstrained analytic possibilities are combined with an ideological agenda, we have a particularly toxic mix, Consider, for instance, an analysis based on a publicly available database that purported to show a link between vaccination and autism, but only in African–American boys who were vaccinated in a particular time window [14]. Not only does this look like classic *p*-hacking, but the analysis also failed to account for important confounders, such as higher immunization levels among children attending special education facilities. Set against a mountain of null findings from other studies, the association was unconvincing, and the paper was subsequently retracted for numerous methodological flaws and conflict of interest. Nevertheless, it caused a flurry of excitement among anti-vaccination activists, some of whom believed that the author had revealed a conspiracy to cover up the truth (http://www.snopes.com/medical/disease/cdcwhistleblower.asp). Even though reputable researchers quickly detected the problems with the study, it had the potential to cause damage by encouraging parents not to vaccinate their children. In order to determine whether a reported association is believable, we need to be able to ‘map the space of what analyses could have been performed’ [15]—which may be unknowable if datasets that did not confirm the researcher's views were never mentioned.

## 6. Proposals to avoid unintended consequences

The issues surrounding research with human participants can mostly be dealt with by anticipating them. This means having the appropriate consents from participants for data-sharing and scrupulously attending to anonymization [10,16]. Guidance by the Medical Research Council (MRC) recommends that researchers who use patient data should set up data-sharing agreements for secondary use; these specify who the data can be shared with and what they will do with it [10].

A good way to keep investigators honest and prevent unwitting bias in analysis is to mask some of the coding: for instance, in an intervention trial, researchers could be kept blind as to which group was which until the analysis was complete. (For a mischievous early example, see http://www.badscience.net/2010/04/righteous-mischief-from-archie-cochrane/.) As MacCoun and Perlmutter noted, blind data analysis is widely adopted in physics as a way of avoiding experimenter bias, yet is virtually unknown in the biomedical sciences [17]. They recommended that bias in analytic decisions can be avoided if detection of outliers and data analyses are decided using datasets that have been perturbed in various ways (e.g. scrambling or shuffling conditions, or adding noise to the data); analysis of the true dataset is conducted only after the full analytic pipeline is determined.

Among those interested in reproducibility, there has been much discussion of the potential of study pre-registration for preventing *p*-hacking and publication bias [18]. Yet in pushing for open data, we have largely overlooked the parallel problem that can occur with secondary data analyses where the goal is not just to replicate the original analysis, or to include the data in a meta-analysis, but to test new hypotheses that go beyond the original aims of the study. In a recent comment, Lewandowsky & Bishop [19] mentioned the possibility of requiring pre-registration for secondary data analyses. Our focus was on controversial areas of research, where researchers were reluctant to release data because they did not trust the impartiality of those who wished to do fresh analyses, but if the principle were more widely applied, it could avoid *p*-hacking and publication bias in secondary analyses more generally. The difficulty, of course, is that if data are already collected, then there are logistic problems in pre-registration of analyses, and this may also seem unduly onerous in many experimental contexts. However, pre-registered analyses could give more confidence in findings when dealing with large, multivariable datasets.

An example of how this can work in practice is the Avon Longitudinal Study of Parents and Children (ALSPAC), a valuable resource for studies of epidemiology and genetics, with high-quality data on a cohort of children and their parents, including DNA, prenatal variables, environmental background and a host of information from questionnaires and, in a subset, direct testing, at several time-points during development [20]. The ALSPAC dataset incorporates over 60 000 variables. In line with procedures recommended by the MRC [10], researchers who wish to set up a data access agreement must state their aims and hypotheses and describe the relevant exposure, outcome and confounder variables that will be used (http://www.bristol.ac.uk/alspac/researchers/access/).

Pre-specification of analyses is suitable when there is a clear advance hypothesis, but scientific progress also depends on serendipitous discoveries from open-ended exploration. Exploratory analyses are to be encouraged, provided it is recognized that their use is for generating hypotheses that should then be tested on a new dataset. With large datasets, this can be achieved by using a random half of the data as a discovery sample and the other half for replication, or by confirming the pattern of results in another dataset.

## 7. Open data where there is a breakdown of trust

Data access agreements with clearly specified hypotheses would not only enhance reproducibility of findings, but could also make researchers working in controversial areas more willing to engage with critics. There may still, however, be concerns about having the same people who collected the data also having a gatekeeper role. What appears to those who did the study as a safeguard against misuse of data can be interpreted as attempts to hide inconvenient results by those who have doubts about a study. The MRC guidelines anticipate this to some extent by requiring that researchers have independent advice and oversight of access agreements.

Where there is a total breakdown of trust between those who collected the data and those who seek to analyse it, there would be value in having an independent adjudicator: essentially, a neutral body with statistical expertise that could help specify the analyses to be done, and ensure that they are done competently. This idea is similar to that behind Berkeley Earth, a non-profit organization that was set up to provide independent and transparent analysis of land temperature records. Their results were far from being a foregone conclusion: one of the founders had started the project because he had reservations about the quality of climate data, and climate sceptics initially expressed full confidence in the methods. However, when Berkeley Earth analyses confirmed the extent of global warming [21], the results were rejected by some critics, and complaints were made about the methodology. Despite this, the project can be deemed a success, insofar as it made it easier to distinguish critics who are interested in evidence, and those for whom no evidence against their position will be sufficient [19]. My proposal is for something more general than Berkeley Earth: an independent statistics and methods unit that could take a fresh look at existing data on any topic where there is substantial public concern that the findings may be flawed. Such an enterprise would require independent funding, but could be justified if it improved public confidence in science.
